# Mechanism-based approach using a biomarker response to evaluate tocilizumab subcutaneous injection in patients with rheumatoid arthritis with an inadequate response to synthetic DMARDs (MATSURI study)

**DOI:** 10.1002/jcph.185

**Published:** 2013-10-12

**Authors:** Shuji Ohta, Tomomi Tsuru, Kimio Terao, Seiji Mogi, Midori Suzaki, Eisuke Shono, Yoshimasa Ishida, Eriko Tarumi, Masato Imai

**Affiliations:** 1 Taga General HospitalIbaraki, Japan; 2 Oasis ClinicIbaraki, Japan; 3 Med Co LTA PS ClinicFukuoka, Japan; 4 Chugai Pharmaceutical Co, LtdTokyo, Japan; 5 Shono Rheumatology ClinicFukuoka, Japan

**Keywords:** tocilizumab, subcutaneous injection, pharmacokinetics, CRP, biomarker

## Abstract

A multicenter, open-label, dose-escalation phase 1/2 study was undertaken to evaluate the optimal subcutaneous tocilizumab dose that would result in exposure comparable to the intravenous tocilizumab 8-mg/kg approved dose in patients with rheumatoid arthritis. A pharmacokinetic and biomarker approach was used to estimate the clinical optimal dose regimen of subcutaneous tocilizumab. Safety and efficacy of subcutaneous tocilizumab were assessed as secondary end points. Patients received subcutaneous tocilizumab at 81 mg every 2 weeks (q2w) (n = 8), 162 mg q2w (n = 12), or 162 mg weekly (qw) (n = 12) for 24 weeks. 88% of 162-mg q2w patients and 100% of 162-mg qw patients maintained mean serum trough tocilizumab concentrations of ≥1 µg/mL, and had exposure comparable with the approved intravenous tocilizumab dose of 8 mg/kg; this resulted in normalized C-reactive protein levels and improvement in ACR20/50/70 responses. The most common adverse events were abnormal laboratory results, which were mild in severity. Anti-tocilizumab antibodies were detected in a few patients in the 81-mg q2w and 162-mg qw groups. In conclusion, coupled with efficacy and tolerability results, the appropriate dose of subcutaneous tocilizumab was determined to be 162 mg q2w for Japanese patients.

Rheumatoid arthritis (RA) is a chronic, inflammatory, autoimmune disease characterized by joint damage, functional disability, and increased mortality. The release of cytokines, including tumor necrosis factor α, interleukin 6 (IL-6), and IL-1, induces chronic inflammatory synovitis and mediates joint destruction.^1^,^2^

Currently, C-reactive protein (CRP) level is used clinically as a biomarker of IL-6 activity and inflammation in RA.^4^ After binding to IL-6 receptor (IL-6R), IL-6 stimulates the synthesis of CRP through activation of the Janus kinase signaling pathway.^1^ Elevated IL-6 levels in patients with RA correlate with disease activity. Because CRP levels are regulated by IL-6, elevated IL-6 levels increase CRP levels following inflammation, and the CRP level reflects the severity of inflammation. Although both IL-6 and CRP levels can be measured, CRP is more reflective of the physiological and inflammatory state of the disease because it is an acute-phase reactant directly responsible for the inflammation process. Tocilizumab is a humanized monoclonal antibody that inhibits IL-6 signaling, including production of CRP.^2^ In patients with RA, tocilizumab treatment normalizes CRP levels as long as the free serum tocilizumab concentration remains ≥1 μg/mL.^4^ This suggests that CRP levels are a useful biomarker of tocilizumab activity.

Some patients with RA prefer self-injectable subcutaneous (SC) formulations of RA therapeutics, such as etanercept and adalimumab, that can be administered at home.^5^–^10^ The main reasons patients prefer SC formulations are reduced outpatient costs and time and reduced hospital treatment time, which can also be beneficial for healthcare professionals.^11^ In Japan, tocilizumab administered by intravenous (IV) infusion at 8 mg/kg is approved for the treatment of patients with RA, polyarticular juvenile idiopathic arthritis, systemic juvenile idiopathic arthritis, and Castleman disease.^12^,^13^ Phase 3 trials of tocilizumab with traditional (synthetic) disease-modifying antirheumatic drugs (DMARDs) as combination therapy or as monotherapy have demonstrated improvements in clinical symptoms, inhibition of radiographic progression, and normalization of CRP levels in patients with RA.^15^–^21^ A self-injectable SC formulation of tocilizumab would provide a further treatment option to patients with RA.

The objective of this phase 1/2 study (MATSURI) was to evaluate the SC tocilizumab dose that resulted in exposure comparable to that achieved with IV tocilizumab in patients with RA. Safety and efficacy of SC tocilizumab were assessed as secondary end points. For identification of the optimal dose of SC tocilizumab, a pharmacokinetic/pharmacodynamic (PK/PD) modeling and simulation approach was used. PK/PD-based modeling has been particularly useful in drug development programs for estimating exposure-response relationships, predicting multiple-dose profiles from a single dose, simulation of phase 2 trials, and formulation development. A modeling and simulation approach would establish whether an SC tocilizumab formulation has a favorable PK profile and effectiveness similar to IV tocilizumab without necessitating additional phase 2 studies.^22^–^23^ We present the results of clinical trial simulations of concentrations of SC tocilizumab 162 mg every 2 weeks (q2w) as well as the efficacy, safety, PK, and PD of multiple-dose regimens of SC tocilizumab.

## Methods

### Study Design

The MATSURI study was a multicenter, open-label, dose-escalation study conducted in Japan with centralized enrollment (pain assessment was conducted in a single-blind method) in Japanese patients with active RA. The investigational review boards of PS Clinic and Hitachi Taga General Hospital (Ibaraki, Japan) approved the study protocol. All patients gave their written informed consent.

The study was conducted in three groups of patients with RA. Patients received SC tocilizumab 81 mg q2w, 162 mg q2w, or 162 mg weekly (qw); cohorts transitioned to the next dose step upon confirmation of tolerability at the third week after the start of treatment. Injections were given in the abdominal region for 5 seconds by a healthcare professional (Figure S1). The study schedule for the SC tocilizumab 81 mg q2w and 162 mg q2w groups was divided into three periods: period I, during which safety, PK, and pain of injection were assessed 3 weeks after 1 dose of SC tocilizumab; period II, during which safety was assessed after three doses of SC tocilizumab were administered at 2-week intervals; and period III, during which patients received 24 weeks of treatment, and safety and PK was assessed throughout. The study schedule for the SC tocilizumab 162 mg qw group involved only periods II and III (period I assessed the single-dose safety, tolerability, and PK for both the q2w and qw regimens). Cohort transition from 81 mg q2w to 162 mg q2w occurred when all eight patients in the 81-mg q2w group demonstrated tolerability up to the third week after the start of treatment with SC tocilizumab (period I; Figure S2). Similarly, cohort transition from 162 mg q2w to 162 mg qw occurred when all 12 patients demonstrated tolerability at the third week after the start of treatment with SC tocilizumab (period I). Cohort transition to the next dose proceeded if <2 patients in each treatment group experienced the same grade ≥3 adverse event (AE). Cohort transition to the 162-mg q2w and 162-mg qw groups proceeded only after a tolerability review meeting was held to determine the suitability of transition to each dose.

In period III, patients in the 81-mg q2w group could have dosing frequency increased to weekly if there was an inadequate antirheumatic response, as determined based on the signs and symptoms of RA, including tender joint count (TJC), swollen joint count (SJC), and CRP levels. The dosing interval for patients in the 162-mg qw group could be decreased if there was an adequate antirheumatic response. Rheumatic response was assessed by categorical score defined by treatment recommendations from both the American College of Rheumatology (ACR) and the European League Against Rheumatism (EULAR).^24^–^25^

Concomitant use of low-dose oral glucocorticoids (≤10 mg/day prednisolone equivalent) was permitted. Intra-articular injections of glucocorticoids and hyaluronate were avoided if at all possible. Other treatments for concurrent disease that were unlikely to affect the efficacy evaluation of SC tocilizumab were permitted.

### Patient Population

Eligible patients in Japan were aged 20–75 years with RA for ≥6 months, as diagnosed using the 1987 criteria of the ACR for the classification of RA.^26^ Additional inclusion criteria were as follows: inadequate response to any synthetic DMARD or immunosuppressive agent, erythrocyte sedimentation rate (ESR) of ≥30 mm/h, or CRP levels ≥1.0 mg/dL.

Exclusion criteria included active tuberculosis (patients undergoing prophylactic chemotherapy for latent tuberculosis infection could participate), serious allergies, active hepatitis B or C infection, or class IV Steinbrocker functional activity within 4 weeks of treatment. Patients were also excluded if they had been treated previously with tocilizumab by either infusion or SC administration; had been treated previously with leflunomide, infliximab, etanercept, or adalimumab within 6 weeks prior to tocilizumab treatment; had received plasmapheresis, surgical procedures, or dose changes in synthetic DMARDs or immunosuppressants within 4 weeks of tocilizumab treatment; had received oral glucocorticoids at a dose >10 mg/day prednisolone equivalent or had a dose increase, new administration, or IV or intramuscular injections of glucocorticoids within 2 weeks of tocilizumab treatment.

### Sampling Strategy With PK and PD Endpoints

The PK model was assumed to be a 2-compartment model with Michaelis–Menten elimination and first-order absorption route. Apparent absorption rate and absolute bioavailability (K_a_ and F) were derived from this study, and other PK parameters were estimated with fixed-disposition PK parameters as previously defined.^27^ Serum tocilizumab concentrations following SC administration q2w were simulated based on estimated-absorption PK parameters and fixed-disposition PK parameters. Simulation of PK and PK/PD analysis was performed using NONMEM (ver7.2, ICON Development Solution, Dublin,Ireland).

The primary PK end point was the serum tocilizumab concentration. The PD end points were anti-tocilizumab antibody, IL-6, and soluble IL-6R (sIL-6R) concentrations. Patients who received SC tocilizumab at least once were included in the PK analysis population. The 81-mg q2w and 162-mg q2w groups were analyzed before each dose and 4, 8, 12, 24, 36, 48, 56, 168, 216, 336, and 384 hours after the first dose. The 162-mg qw group was analyzed before the first, second, third, fourth, and fifth injection time points; every 2 weeks from the fifth dose; and at the last observation. If the serum tocilizumab concentration fell below the lower limit of quantification (LLOQ), 0.1 µg/mL, a value of 0.05 was imputed.

CRP levels were measured in the 81-mg q2w and 162-mg q2w groups at enrollment; before each dose; 4, 8, 12, 24, 36, 48, 56, 168, 216, 336, and 384 hours after the first dose; and on the last observation day (withdrawal). CRP levels were measured in the 162-mg qw group at enrollment; before the first, second, third, fourth, and fifth doses of the investigational product; every 2 weeks from the fifth dose; and on the last observation day (withdrawal). CRP was measured at SRL Inc. (Tokyo, Japan). The assessment between serum concentration and CRP was for time points after 168 hours. Times points after 168 hours are >5 times greater than the t_1/2_ of CRP (46.4 ± 21.7 hours).^28^ The signal blockade of TCZ was assumed to start at immediately after treatment (0 hour), and therefore the de novo production of CRP would have ceased. However, in order to assess de novo CRP production using serum levels of CRP, the elimination rate of CRP (1 week) must be taken into account so time points taken before 168 hours interval were not included.

### Efficacy

The primary efficacy end points were CRP levels and ESR at the completion of period I, period II, period III, and over the total time in the per-protocol population. The per-protocol population included patients in the intent-to-treat population, except for those with protocol violations or those who withdrew early. Secondary end points included ACR response rates of 20%, 50%, and 70% (ACR20, ACR50, and ACR70, respectively), the disease activity score using 28 joints (EULAR disease activity score [DAS28]) and simplified disease activity index (SDAI).^29^ The relationship between serum tocilizumab concentrations and CRP was also evaluated. The last observation carried forward method was used to impute missing data for the ACR core set components and the parameters derived from the components (ACR20, ACR50, ACR70, DAS28, and clinical disease activity index [CDAI]).^30^

### Safety

The primary safety end points were the incidence and severity of AEs and adverse drug reactions. Secondary safety end points were clinical symptoms, physiological tests, 12-lead electrocardiogram, and laboratory test values. AEs and serious AEs were classified using the Medical Dictionary for Regulatory Activities, version 11.1. The number of patients who experienced AEs and the total number of AEs were tabulated by event and defined using system organ class and preferred term (Common Terminology Criteria for Adverse Events v 3.0). An absorption test was performed for each measurement to confirm positive results obtained by the screening method or measurement of neutralizing antibodies or immunoglobulin (Ig) E antibodies.

### Injection-Site Pain

Assessment of injection-site pain was conducted as a single-blind crossover between SC physiological saline and SC tocilizumab for dosing order and injection site (right or left abdomen). The difference in injection-site pain with SC physiological saline and SC tocilizumab was evaluated in the 81-mg q2w and 162-mg q2w groups at the time of dosing in period I. The volume of SC tocilizumab or physiological saline in the respective syringes was 0.45 mL for the 81-mg q2w group and 0.90 mL for the 162-mg q2w group. A syringe and 24 G needle were used to administer SC tocilizumab and physiological saline; the same physician administered the SC tocilizumab and physiological saline into the left and right abdomen of patients for 5 seconds, with 5 minutes between injections. The patients assessed their level of pain after each individual injection and compared their pain between the first and second injections on the basis of the six levels of the Wong-Baker FACES Pain Rating Scale and using a 10-cm visual analog scale (VAS).

Scores were given for each level of the Wong-Baker FACES Pain Rating Scale (Figure S3), and analysis of variance was used to determine differences between groups for the order of administration, injection site, and drug preparation as the fixed-effect factors and the patient as the random-effect factor. The least-squares means and 95% CIs of the differences between SC tocilizumab and physiological saline were calculated. The least-squares means by level of each fixed-effect factor and the least-squares means and 95% CIs of the differences between SC tocilizumab and physiological saline were calculated and compared.

### Serum Tocilizumab Concentration

An enzyme-linked immunosorbent assay (ELISA) was developed to detect tocilizumab in humans. This ELISA is based on immobilization of sIL-6R (SR-344, 1 µg/mL; Chugai Pharmaceutical Co, Ltd, Tokyo, Japan) onto microtiter plates to capture tocilizumab in the samples. Tocilizumab is detected by a digoxigenin (DIG)-labeled anti-tocilizumab secondary antibody (Chugai Pharmaceutical Co, Ltd.), which is detected by an anti-DIG horseradish peroxidase (HRP)–conjugated antibody that reacts with its substrate. The colorimetric reaction was quantified by measuring the absorbance at 405 nm (with 490 nm as reference) using a microplate reader. The concentration of free tocilizumab in the specimen was calculated from a calibration curve prepared from standard solutions. The LLOQ of the ELISA was validated at 0.1 µg/mL, the limit of detection at 0.025 µg/mL and the upper limit of quantification at 3.2 µg/mL. In addition, the linearity of dilution was also confirmed up to 400-fold. All serum samples were used in the assay within 4 weeks and stored below −20°C. The assays were validated and performed at SRL Inc.

### Immunogenicity

Screening and measurement of neutralizing antibodies and IgE antibodies were used to test for anti-tocilizumab antibodies. All samples were tested in parallel in screening and confirmation, antibody fragment (Fab), and IgE assays. The anti-tocilizumab antibody screening and confirmation assay consisted of a sandwich-type ELISA with an additional competitive displacement step for the confirmation assay. The ELISA used tocilizumab immobilized on microtiter plates for the capture of anti-tocilizumab antibodies complexed with preincubated DIG-labeled tocilizumab. The bound complex of anti-tocilizumab antibody and DIG-labeled tocilizumab was detected by an HRP-conjugated anti-DIG antibody that reacted with its substrate 2,2′-azino-bis(3-ethylbenzothiazoline-6-sulphonic acid) (ABTS) and gave a subsequent photometric readout. The positive/negative cut point was determined at the 95% CIs using the optical density from multiple analyses of serum samples from healthy volunteers or patients with RA who had not received tocilizumab. Separate cutoffs were established for patients with RA.

Confirmation of a positive screening result (differentiation between nonspecific and specific binding) was performed by means of a displacement reaction step, using the same method except that the preincubation stage of the assay also included an inhibitory bulk of unlabeled tocilizumab with the anti-tocilizumab antibody and DIG-labeled tocilizumab. If the decrease in absorbance due to the presence of tocilizumab was <20%, the test result was considered negative. If the decrease in absorbance was ≥20%, the test result was considered positive.

A Fab assay was used to test for the presence of anti-tocilizumab antibodies using a bridging enzyme immunoassay with Fabs of tocilizumab. This assay is specific for human anti-human antibodies directed against the antigen-binding part of tocilizumab. Anti-tocilizumab antibodies are captured by the tocilizumab Fab and detected by biotinylated tocilizumab Fab (secondary antibody), avidin-labeled peroxidase, and its substrate *o*-phenylenediamine dihydrochloride. The Fab assay was validated and the LLOQ set at 3.91 ng/mL using a rabbit polyclonal anti-tocilizumab antibody as a positive standard.

The assay used to test for the presence of anti-tocilizumab IgE antibodies in human serum is based on the commercially available ImmunoCAP assay system (UniCAP 1000 Specific IgE; Phadia AB, Uppsala, Sweden). CAPs with bound allergen (tocilizumab), calibration CAPs, and CAPs with bound house dust mite (*Dermatophagoides pteronyssinus*) allergen (for quality control purposes) were used. Study samples were incubated with the tocilizumab CAPs, calibration samples with the calibration CAPs, and quality control samples with the house dust mite CAPs. The CAPs were allowed to react with any specific IgE antibody in the serum, and excess serum components were removed by washing. IgE antibody that was bound to the ImmunoCAP allergens was reacted with β-d-galactosidase—labeled human antihuman IgE antibody. Subsequent addition of a fluorogenic substrate (4-methylumbelliferyl-β-d-galactopyranoside) led to the generation of fluorescent product. The reaction was terminated, and the antibody concentration was estimated by comparison with the fluorescence intensity of the calibration samples. All the assays were validated and performed at SRL Inc.

## Results

### Patient Disposition

**Figure 1:**
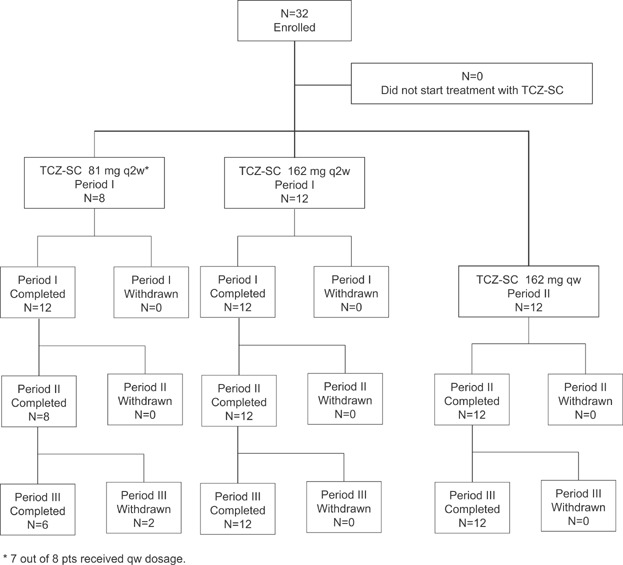
Patient disposition over periods I, II, and III.

This study enrolled 32 patients (SC tocilizumab 81 mg q2w [n = 8]; SC tocilizumab 162 mg q2w [n = 12]; SC tocilizumab 162 mg qw [n = 12]; Figure 1). No patients withdrew in period I or II. In period III, two patients withdrew in the 81-mg q2w group: one because of decreased white blood cell count and neutrophil levels and another because of an insufficient therapeutic response. In the 81-mg q2w group, the dosing frequency was increased to qw in seven patients in period III (1 patient withdrew from the study). In the 162-mg qw group, the dosing frequency was decreased to q2w at least once in four patients in period III. In three of these patients, the dosing frequency was decreased to q2w or every 3 weeks only once as a result of AEs; one patient's dosing frequency was decreased to q2w and was maintained at q2w until the end of study as he maintained sufficient efficacy.

### Baseline Demographics and Disease Characteristics

Patient demographics and clinical characteristics were similar between the groups (Table1). The mean weight, height, and age of patients were similar between the 81-mg q2w, 162-mg q2w, and 162-mg qw groups. The mean ± standard deviation (SD) durations of RA were 5.3 ± 6.3, 8.0 ± 9.5, and 9. ± 6.5 years in the 81-mg q2w, 162-mg q2w, and 162-mg qw groups, respectively. Glucocorticoids were received by 75%, 58%, and 42% of patients in the 81-mg q2w, 162-mg q2w, and 162-mg qw groups, respectively, at the start of treatment with SC tocilizumab.

**Table 1:** Patient Characteristics at Baseline (Safety Population)^a^

	SC Tocilizumab 81 mg q2w (n = 8)	SC Tocilizumab 162 mg q2w (n = 12)	SC Tocilizumab 162 mg qw (n = 12)
Male, n (%)	0	4 (33)	6 (50)
Female, n (%)	8 (100)	8 (67)	6 (50)
Age, years	55 ± 14	59 ± 10	52 ± 14
Body weight, kg	53.5 ± 13.3	55.5 ± 10.6	58.9 ± 12.5
Disease duration, years	5.3 ± 6.3	8.0 ± 9.5	9.3 ± 6.5
RF positive, n (%)	7 (88)	9 (75)	8 (67)
SJC (in 66 joints)	6.8 ± 2.4	11.8 ± 4.3	15.4 ± 6.3
TJC (in 68 joints)	7.5 ± 3.1	7.3 ± 7.0	7.3 ± 8.0
JHAQ score	1.5 ± 0.8	1.0 ± 0.7	0.9 ± 0.8
Patient's pain assessment, mm	55.8 ± 16.4	59.4 ± 20.8	51.8 ± 33.5
Patient's global assessment, mm	58.1 ± 15.3	58.1 ± 19.0	53.6 ± 32.4
Physician's global assessment, mm	45.0 ± 12.1	64.1 ± 24.5	67.7 ± 14.9
CRP, mg/dL	3.0 ± 2.1	3.1 ± 3.3	3.4 ± 4.5
ESR, mm/h	74.0 ± 28.8	71.2 ± 28.5	57.1 ± 35.9
DAS28-ESR	5.4 ± 0.5	5.6 ± 1.0	5.3 ± 1.3
DAS28-CRP	4.5 ± 0.5	4.7 ± 1.2	4.7 ± 1.5
Oral glucocorticoids administered, n (%)	6 (75)	7 (58)	5 (42)
Dose, mg/day	3.3 ± 2.2	2.1 ± 2.1	2.8 ± 3.9
Previous methotrexate, n (%)	8 (100)	11 (92)	12 (100)
Maximum dose, mg/week	6.8 ± 1.0	8.2 ± 3.8	9.3 ± 2.4
Previous biologic agent, n (%)	1 (13)	2 (17)	3 (25)

CRP, C-reactive protein; DAS28, disease activity score using 28 joints; ESR, erythrocyte sedimentation rate; JHAQ, Japanese version of the Health Assessment Questionnaire; q2w, every other week; qw, weekly; RF, rheumatoid factor; SC, subcutaneous; SJC, swollen joint count; TJC, tender joint count.

a aData are mean ± SD unless otherwise noted.

### Pharmacokinetics: Simulation Prior to Initiation

**Figure 2:**
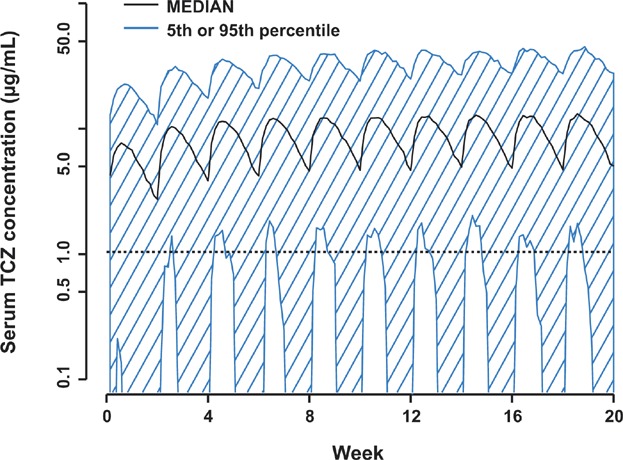
Simulated time course of serum tocilizumab concentrations for 20 weeks in the subcutaneous tocilizumab 162 mg every 2 weeks group. The broken line represents a serum tocilizumab level of 1 µg/mL.

The simulated time course of serum tocilizumab concentration for the SC tocilizumab 162-mg q2w group is shown in Figure 2; 81% of patients maintained a serum tocilizumab concentration >1 µg/mL at trough level.

### Pharmacokinetics: Analysis

The PK modeling parameters are provided in Table S1.

**Figure 3:**
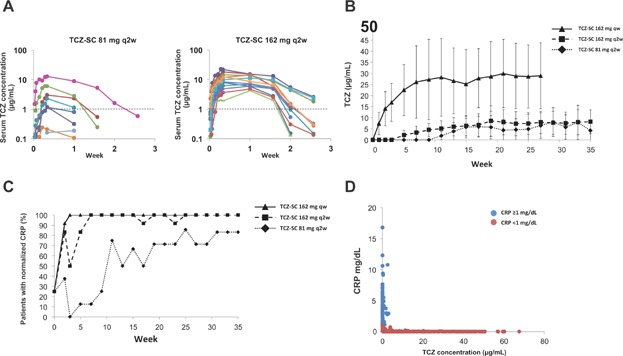
(A) Individual serum trough tocilizumab (TCZ) concentrations for patients receiving a single subcutaneous (SC) injection of TCZ at 81 mg every 2 weeks (q2w) or 162 mg q2w. (B) Mean serum trough TCZ concentrations for patients receiving multiple injections of TCZ SC at 81 mg q2w, 162 mg q2w, or 162 mg weekly (qw). (C) Percentage of patients achieving normalized C-reactive protein (CRP) levels over time after receiving injections of TCZ SC at 81 mg q2w, 162 mg q2w, or 162 mg qw. (D) The relationship between serum TCZ and CRP levels.

Individual serum tocilizumab concentrations for patients in the 81-mg q2w and 162-mg q2w groups were evaluated in period I after a single dose of SC tocilizumab (Figure 3A). In the 81-mg q2w group, the serum tocilizumab concentration was above the LLOQ (0.1 µg/mL) in 100% of patients for 7 days after administration. The serum tocilizumab concentration in the 81-mg q2w group was below the LLOQ in 63% of patients at day 7 and for all patients 10 and 21 days after administration, respectively. One patient in the 81-mg q2w group had a tocilizumab concentration ≥1 µg/mL 14 days after administration. The mean ± SD maximal concentration (C_max_) was 3.4 ± 4.3 µg/mL after the initial SC dose of 81 mg as determined by noncompartmental analysis. The area under the concentration curve (AUC) was 21.4 ± 33.3 µg day/mL, and the time to maximal concentration (t_max_) ranged from 1.5 to 6.0 days. The serum tocilizumab concentration was too low to evaluate the elimination phase after the initial 81-mg SC dose.

In the 162-mg q2w group, the serum tocilizumab concentration was above the LLOQ in all patients up to 14 days after administration. Eight patients in the 162-mg q2w group had tocilizumab concentrations ≥1 µg/mL 14 days after administration. The serum tocilizumab concentration was below the LLOQ in 33% of patients 17 days after administration and in 75% of patients 21 days after administration. The mean ± SD C_max_ was 10.9 ± 5.6 µg/mL after the initial SC dose of 162 mg as determined by noncompartmental analysis. The AUC was 96.7 ± 53.7 µg day/mL, and the t_max_ ranged from 2.0 to 7.2 days. The elimination half-life was 1.6 ± 0.24 days after the initial 162-mg SC dose.

The serum trough tocilizumab concentrations were also evaluated for all studied SC tocilizumab doses (Figure 3B). The serum trough tocilizumab concentration was below the LLOQ through week 9 for 88% of patients in the 81-mg q2w group. Although serum trough tocilizumab concentrations were detected from week 11, the 81-mg q2w group was not considered to be evaluable at that time because the dosing frequency had increased to qw injections for seven patients. Of these patients who had increased to qw, serum trough tocilizumab concentrations after week 9 were similar to those of the 162-mg q2w group. Serum trough tocilizumab concentrations increased and reached steady state by week 15 in the 162-mg q2w and 162-mg qw groups; mean trough tocilizumab concentrations ranged from 6 to 9 µg/mL and from 25 to 30 µg/mL, respectively.

### CRP and ESR

In the 81-mg q2w group, the CRP normalization rate (patients achieving levels <1 mg/dL) was 2/8 patients (25%) at the end of period II, immediately prior to the next dose (dose interval q2w; Figure 3C). Although the normalization rate increased over time, 10/12 (83%) patients in the 81-mg q2w group achieved normalized CRP levels by the last observation at week 35 (during period III, the dose interval was qw). All of the patients in the 162-mg q2w group achieved normalized CRP levels by week 7 and subsequently maintained normal levels. All of the patients in the 162-mg qw group achieved normalized CRP levels by week 3 and subsequently maintained normal levels.

A trend of improvement in CRP levels was observed with increasing serum trough tocilizumab concentrations (Figure 3D). Most patients who maintained a trough tocilizumab concentration of ≥1 µg/mL had CRP levels that were <1 mg/dL.

The ESR normalized in none of the patients in the 81-mg q2w and 162-mg q2w groups at week 3 and in 9/12 (75%) patients in the 162-mg qw group (Figure S4). At the last observation (week 35 in the 81-mg q2w and 162-mg q2w groups and week 28 in the 162-mg qw group), the ESR normalized in 4/6 (67%) patients in the 81-mg q2w group, 10/12 (83%) of patients in the 162-mg q2w group, and 8/12 (67%) patients in the 162-mg qw group. A trend of improvement in ESR was observed with increasing serum trough tocilizumab concentrations.

### Clinical Efficacy

**Figure 4:**
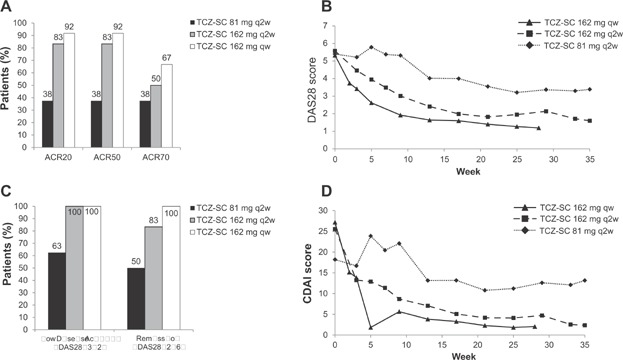
(A) American College of Rheumatology response rates of 20%, 50%, and 70% (ACR20, ACR50, and ACR70, respectively) at week 25 in patients receiving subcutaneous injections of tocilizumab (TCZ SC) at 81 mg every 2 weeks (q2w), 162 mg q2w, or 162 mg weekly (qw). (B) Disease activity score using 28 joints (DAS28) over time in the three groups. (C) Low disease activity (DAS28 ≤ 3.2) and remission (DAS28 < 2.6) at week 25. (D) Clinical disease activity index (CDAI) score over time.

In the 81-mg q2w group, the ACR20 response rate was 13% (1/8 patients) through week 9, and no patients in this group achieved an ACR50 response. After the dosing frequency was increased to qw at week 9 in the 81-mg q2w group, the ACR20 and ACR50 response rates increased and reached maximum response rates of 50% (4/8) and 38% (3/8), respectively. ACR20 and ACR50 response rates did not differ significantly between the 162-mg q2w and 162-mg qw groups after week 7 (Figure 4A). The ACR20 response rate increased to 83% (10/12 for both groups) at week 13 in both groups. The ACR50 response rate increased to 75% (9/12) by week 17 in the 162-mg qw group and to 83% (10/12) by week 21 in the 162-mg q2w group and remained at this level until the last observation. The ACR20, ACR50, and ACR70 response rates were similar between the 162-mg q2w and 162-mg qw groups at week 25.

In the 81-mg q2w group, DAS28 scores did not change from baseline through week 9 but improved from week 13 onward (Figure 4B); the mean DAS28 score remained constant at approximately 3.3 from week 25. In the 162-mg q2w and 162-mg qw groups, the DAS28 score improved over time from week 3 and week 2, respectively. The mean DAS28 score was 1.60 at week 35 in the 162-mg q2w group and 1.19 at week 28 in the 162-mg qw group.

In the 81-mg q2w group, the proportions of patients who achieved DAS28 remission (DAS28 < 2.6) or low-disease activity (LDA; DAS28 ≤ 3.2) at week 25 were 63% (5/8) and 50% (4/8), respectively (Figure 4C). In the 162-mg q2w and 162-mg qw groups, LDA and remission rates were similar at week 25.

CDAI scores decreased with some fluctuation after the start of treatment in the 81-mg q2w group. CDAI scores decreased over time after the start of treatment in both the 162-mg q2w and 162-mg qw groups (Figure 4D). The SDAI followed a trend similar to that of the CDAI in all of the treatment groups.

Only 63% (5/8) of patients had achieved a good or moderate response at week 35 in the 81-mg q2w group. A good EULAR response was achieved by all patients at week 17 in both the 162-mg q2w and 162-mg qw groups.

### Safety

All patients experienced ≥1 AE (Table2). The most common AEs were abnormal laboratory results: elevated concentrations of blood triglycerides, low-density lipoprotein cholesterol, alanine aminotransferase, and aspartate aminotransferase. Adverse drug reactions occurred in all patients in the 81-mg q2w group, 83% of patients in the 162-mg q2w group, and all patients in the 162-mg qw group. The most common adverse drug reactions were increased levels of blood triglycerides (n = 10 patients [31% overall]), low-density lipoprotein cholesterol (n = 8 patients [25%]), alanine aminotransferase (n = 7 patients [22%]), and aspartate aminotransferase (n = 5 patients [16%]); nasopharyngitis (n = 9 patients [28%]); and pharyngitis (n = 5 patients [16%]). No patients in the study died; 1 patient in the 162-mg q2w group had an anaphylactic reaction due to a food allergy.

**Table 2:** Overall Safety Summary

	SC Tocilizumab 81 mg q2w (n = 8)	SC Tocilizumab 162 mg q2w (n = 12)	SC Tocilizumab 162 mg qw (n = 12)
Patients with ≥1 AE, n (%)	8 (100)	12 (100)	12 (100)
No. of AEs	31	55	48
Patients with ≥1 ADR, n (%)	8 (100)	10 (83)	12 (100)
No. of deaths	0	0	0
No. of malignancies	0	0	0
No. of serious AEs	1	0	0
No. of serious ADRs	1	0	0
No. of AEs leading to withdrawal	1	0	0
No. of AEs leading to dose modification/interruption	1	1	3
No. of injection-site reactions	0	1	0
No. of infusion-related reactions/systemic reactions to injection	3	0	1
Infections, n (%)	3 (38)	6 (50)	8 (67)
Laboratory abnormalities, n (%)	6 (75)	9 (75)	10 (83)
Gastrointestinal disorders, n (%)	1 (13)	6 (50)	2 (17)

ADR, adverse drug reaction; AE, adverse event; q2w, every 2 weeks; qw, weekly; SC, subcutaneous.

The rates of infections were 53%, 50%, and 67% in the 81-mg q2w, 162-mg q2w, and 162-mg qw groups, respectively. The most common event was nasopharyngitis. All infections were considered mild in severity, except for 1 serious infection of pyelonephritis in the 81-mg q2w group.

One injection-site hemorrhage occurred in the 162-mg q2w group. The event was mild in severity and improved with treatment. Injection-site pain was evaluated in the 81-mg q2w and 162-mg q2w groups in period I. The analysis, based on the Wong-Baker FACES Pain Rating Scale (Figure S3), demonstrated a mean difference between SC tocilizumab and saline of 0.0 (95% CI, −1.6 to 1.6) in the 81-mg q2w group and 0.33 (95% CI, −0.5 to 1.2) in the 162-mg q2w group. The difference in severity of pain was also analyzed by a VAS assessment of pain. The mean VAS-based differences between SC tocilizumab and saline were −12.4 mm (95% CI, −40.5 to 15.9) in the 81-mg q2w group and 7.9 mm (95% CI, −13.2 to 29.1) in the 162-mg q2w group. No specific trends were identified for either group based on the Wong-Baker FACES Pain Rating Scale or VAS analyses.

### Immunogenicity

Anti-tocilizumab antibodies were detected in three of eight patients in the 81-mg q2w group and in 2 of 12 patients in the 162-mg qw group in the screening assay. IgE antibodies were detected in three patients in the 81-mg q2w group and in two patients in the 162-mg q2w group. Neutralizing antibodies were not detected in any patients. Of patients who developed anti-tocilizumab antibodies, no impact of the antibodies on the efficacy or safety of tocilizumab was observed.

## Discussion

The pharmacodynamics and pharmacokinetics of IV tocilizumab and SC tocilizumab were previously assessed in a separate study with healthy volunteers.^31^ This study was undertaken to support the selection of the optimal SC tocilizumab dose that would result in exposure (C_trough_) comparable to the IV tocilizumab 8 mg/kg approved dose in patients with RA, similar to a Phase 2a study.^32^ A PK and biomarker approach was used to estimate the clinical optimal dose regimen of SC tocilizumab, evaluate the time course of serum trough tocilizumab concentrations, and assess patient variability in maintaining normalized CRP levels. Coupled with efficacy and tolerability results, SC administration of tocilizumab 162 mg q2w resulted in comparable exposure.

A previous study reported that 62% of patients who received a single dose of IV tocilizumab 8 mg/kg every 4 weeks (q4w) maintained the minimum effective serum tocilizumab concentration of ≥1 µg/mL for ≥ 4 weeks after treatment.^33^–^34^ In this MATSURI study, following administration of a single dose of SC tocilizumab 162 mg, 61% of patients had serum trough tocilizumab concentrations ≥1 µg/mL up to 2 weeks after administration compared with 13% of patients who received a single dose of 81 mg. These results were similar to those in the Phase 3 MUSASHI study that evaluated SC tocilizumab 162-mg q2w in Japanese patients with RA and observed 61% of patients had a tocilizumab concentration of ≥1 µg/mL 2 weeks after the first administration. Moreover, changing the SC tocilizumab 162-mg dosing interval from q2w to qw resulted in three to four times higher mean trough tocilizumab concentrations (6–9 vs. 25–30 µg/mL), which are 2 times higher than those in a phase 3 clinical trial (MRA213JP) of IV tocilizumab (9.6–12.7 µg/mL).^34^ Therefore, SC tocilizumab 162 mg q2w will be adopted as the dose of future phase 3 trials.

Previous clinical trials of IV tocilizumab demonstrated that maintaining serum trough tocilizumab concentrations ≥1 µg/mL resulted in a normalized CRP concentration (<1 mg/dL).^4^–^35^ This suggests that CRP is a useful biomarker for tocilizumab levels high enough to inhibit IL-6 signaling. Therefore, CRP can be used as a biomarker for IL-6 inhibition by SC tocilizumab when serum trough concentrations of tocilizumab are ≥1 µg/mL. The present study demonstrated that both the SC tocilizumab 162 mg q2w and 162 mg qw regimens had serum trough tocilizumab concentrations necessary to normalize CRP levels and show an improvement in clinical symptoms, such as in ACR core set parameters (SJC, TJC, painful joint count, pain VAS, and patient's global assessment); however, in the 81-mg q2w group, minimum improvements were observed in clinical symptoms (Figure 4). These findings suggest that SC tocilizumab 162 mg q2w is the lowest dose effective at normalizing CRP levels and improving clinical symptoms in a Japanese population with representative weight distribution; tocilizumab 162 mg qw is also considered a therapeutic dose.

Mode-of-action–based prediction using this PK and biomarker approach was critical in estimating the optimal SC tocilizumab dose. Based on the outcomes measured in this study, 162 mg q2w appeared to offer an acceptable risk/benefit trade-off. PK simulation supported that the rate of serum trough tocilizumab concentrations ≥1 µg/mL for SC tocilizumab 162 mg q2w was similar to those for IV tocilizumab 8 mg/kg q4w. This prediction method can eliminate the need for phase 2b SC tocilizumab trials because the optimal dosing can be determined directly in phase 2. This allows for the faster clinical development of RA therapeutics to phase 3 trials.^22^–^23^

The tolerability of SC tocilizumab at exposures in the range achieved with the IV tocilizumab 8-mg/kg dose was confirmed up to a dose of 162 mg qw. The major AEs in the present study were abnormal laboratory results, although AEs reported in the five Japanese clinical trial long-term extension studies of IV tocilizumab in patients with RA were synovial cyst, intervertebral disc disorder, and contrast media allergy in one patient each.^36^ All of these events resolved without sequelae and were mild in severity. Few patients in this study developed anti-tocilizumab antibodies. Immunogenicity and AEs cannot be adequately studied in this small number of patients.

The lack of pain contributes to the overall treatment experience and may be an important component of a patient's preference for SC therapies. No differences were identified between SC tocilizumab and saline in the assessment of injection-site pain associated with the order of injection by either the Wong-Baker FACES Pain Rating Scale or VAS. One reason for this may be that the pH (≈6.5) and osmotic pressure (1.0) of solution of SC tocilizumab is similar to that of saline. These findings indicate that the SC formulation of tocilizumab 162 mg was well tolerated, with little pain as well as with fewer and milder AEs.

SC tocilizumab injections q2w would be comparable to the frequency of delivery of other approved SC treatments for RA and therefore equally as accessible for patients. Abatacept and etanercept are administered qw. Adalimumab is administered SC q2w but can be administered qw as monotherapy. However, some RA therapeutics are administered less frequently, including golimumab (monthly) and certolizumab pegol (q2w initially followed by q4w for maintenance therapy).

In summary, this study supported the dose selection of the SC tocilizumab dose that results in exposure comparable to that of the approved IV tocilizumab 8-mg/kg q4w dose in the Japanese population. Coupled with efficacy and tolerability results, the appropriate dose of SC tocilizumab was determined to be 162 mg q2w for Japanese patients. An SC tocilizumab formulation would provide an additional administration option and dosing flexibility for patients with RA. Larger clinical trials are needed to confirm the PK, long-term efficacy, and safety of SC tocilizumab.

## Declaration of Conflicting Interests

Shuji Ohta has received fees for participating in speakers bureaus. Kimio Terao, Yoshimasa Ishida, Eriko Tarumi Masato Imai are employees of Chugai Pharmaceutical Co., Ltd. Tomomi Tsuru, Seiji Mogi and Midori Suzaki have no conflicts of interest to declare.

## Funding

This study was funded by Chugai Pharmaceutical Co., Ltd. Support for third-party writing assistance for this manuscript, furnished by Denise Kenski, PhD of Health Interactions, was provided by F. Hoffmann-La Roche, Ltd.
