# Supplementary material for: Mechanism-based approach using a biomarker response to evaluate tocilizumab subcutaneous injection in patients with rheumatoid arthritis with an inadequate response to synthetic DMARDs (MATSURI study)
Source: J Clin Pharmacol. 2013 Oct 12;54(1):109–19. doi: 10.1002/jcph.185 (PMC3908364; doi:10.1002/jcph.185)
Supplement: Supplementary file 1 [file jcph0054-0109-sd1.docx]

**SUPPLEMENTAL DATA**

**Mechanism-Based Approach Using a Biomarker Response to Evaluate Tocilizumab Subcutaneous Injection in Patients With Rheumatoid Arthritis With an Inadequate Response to Synthetic DMARDs (MATSURI Study)**

**Shuji Ohta,^1,2^ Tomomi Tsuru,^3^ Kimio Terao,^4^ Seiji Mogi,^1^ Midori Suzaki,^3^ Eisuke Shono,^5^ Yoshimasa Ishida,^4^ Eriko Tarumi,^4^ and Masato Imai^4^**

**^1^**Taga General Hospital, Ibaraki, Japan; ^2^Oasis Clinic, Ibaraki, Japan; ^3^Med Co LTA PS Clinic, Fukuoka, Japan; ^4^Chugai Pharmaceutical Co, Ltd, Tokyo, Japan; ^5^Shono Rheumatology Clinic, Fukuoka, Japan

**Table S1. Pharmacokinetic parameters derived from the simulation**

| **Parameter** | **Estimate** |
| --- | --- |
| **Fixed Effect** | |
| Ka, /day | 0.156 |
| F | 0.745 |
| CL , L/day | 0.3^a^ |
| Vc, L | 3.5^a^ |
| Q , L | 0.2^a^ |
| Vp, L | 2.9^a^ |
| VM, mg/day, | 7.5^a^ |
| KM, μg/mL | 2.7^a^ |
| **Interindividual variability** | |
| VAR(ηKa), CV% | 38 |
| VAR(ηF), CV% | 26 |
| VAR(ηCL), CV% | 39^a^ |
| VAR(ηV1), CV% | 37^a^ |
| VAR(ηV2), CV% | 66^a^ |
| VAR(ηVM), CV% | 54^a^ |
| COV(ηCL:ηV1), r | 0.6^a^ |
| COV(ηCL:ηV2), r | -0.1^a^ |
| COV(ηCL:ηVM), r | -0.5^a^ |
| COV(ηV1:ηV2), r | 0.5^a^ |
| COV(ηV1:ηVM), r | 0.2^a^ |
| COV(ηV2:ηVM), r | 0.2^a^ |
| **Residual error** | |
| Additive (σprop), μg/mL | 2.4^a^ |
| Proportional (σadd), % | 22^a^ |

CL, clearance; COV, covariance; KM, Michaelis-Menten constant; Q, intercompartmental clearance; r, correlation coefficient; RSE, relative standard error of estimate; σ, standard error; Vc, central volume of distribution; Vp, peripheral volume of distribution; VAR, variance; VM, maximum elimination rate.

^a^ Estimate was referenced previously.^25^

**Figure S1. Study Schedule**


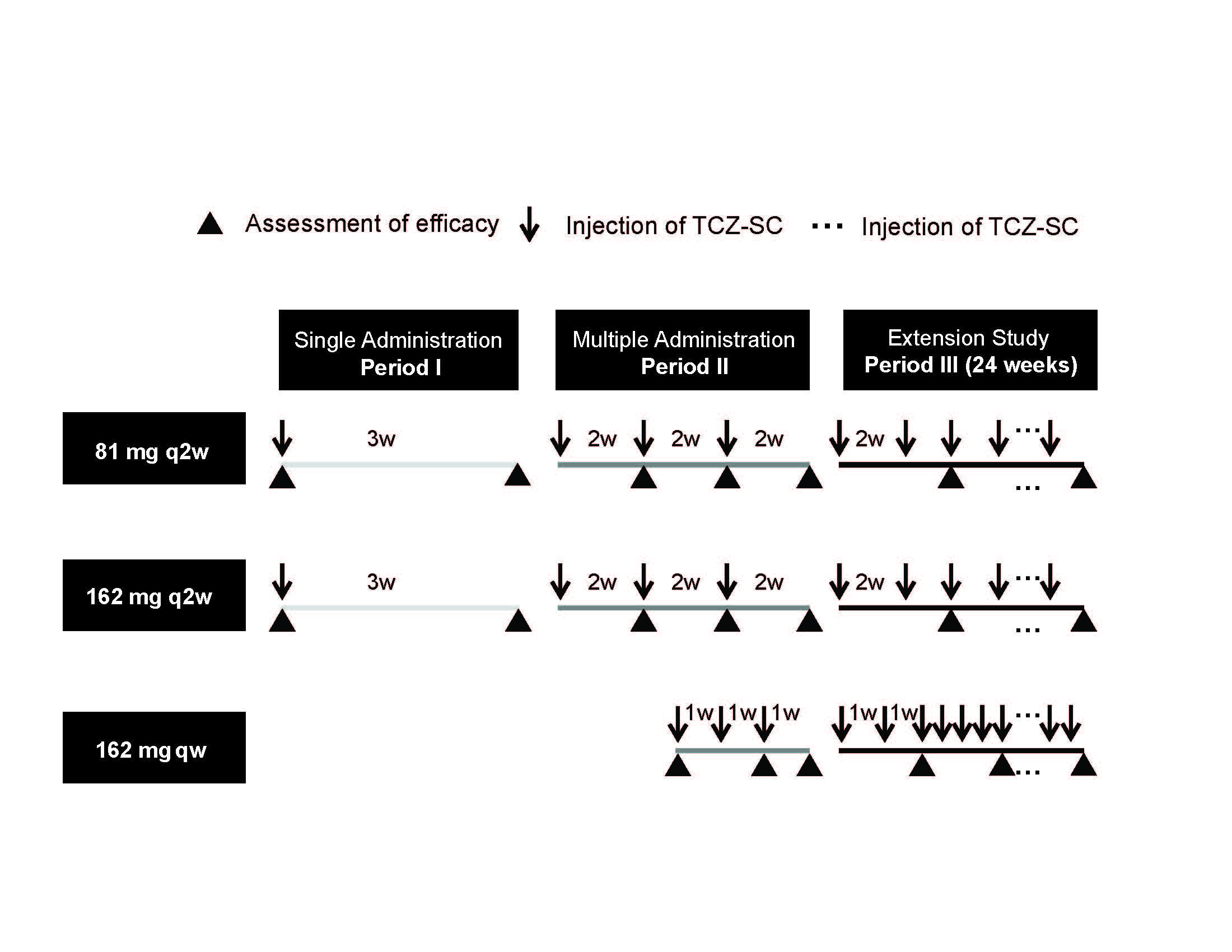


q2w, every 2 weeks; qw, weekly; TCZ-SC, subcutaneous tocilizumab.

**Figure S2. Flow of Cohort Transition**


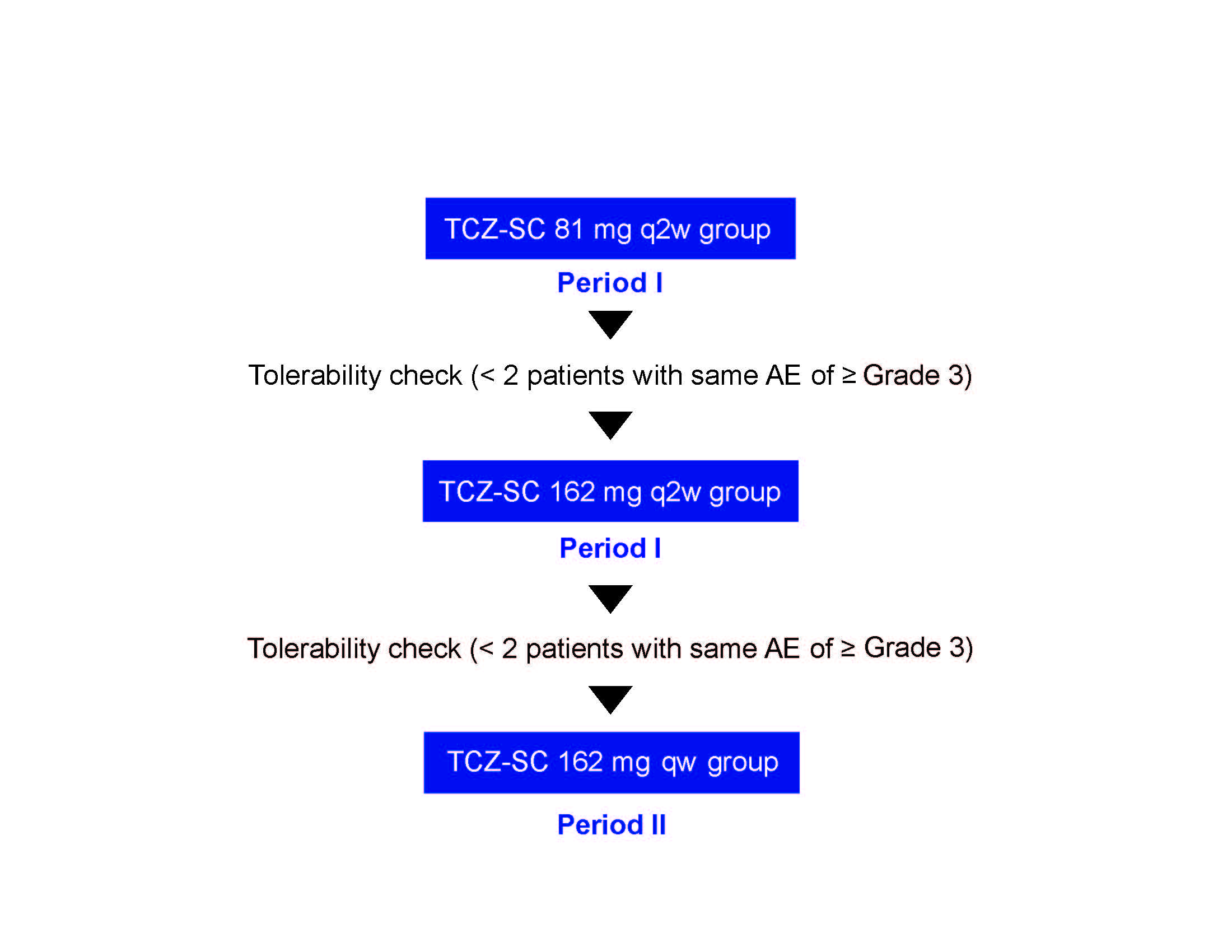


AE, adverse event.

**Figure S3. Patients were assessed for the level of localized irritation (level of pain) at the injection site based on the following 6 levels of the Wong-Baker FACES Pain Rating Scale.**

0. No hurt (extremely happy owing to a complete lack of pain)

1. Hurts a little bit

2. Hurts a little more

3. Hurts even more

4. Hurts a whole lot

5. Hurts worst (cannot imagine hurting more than this)


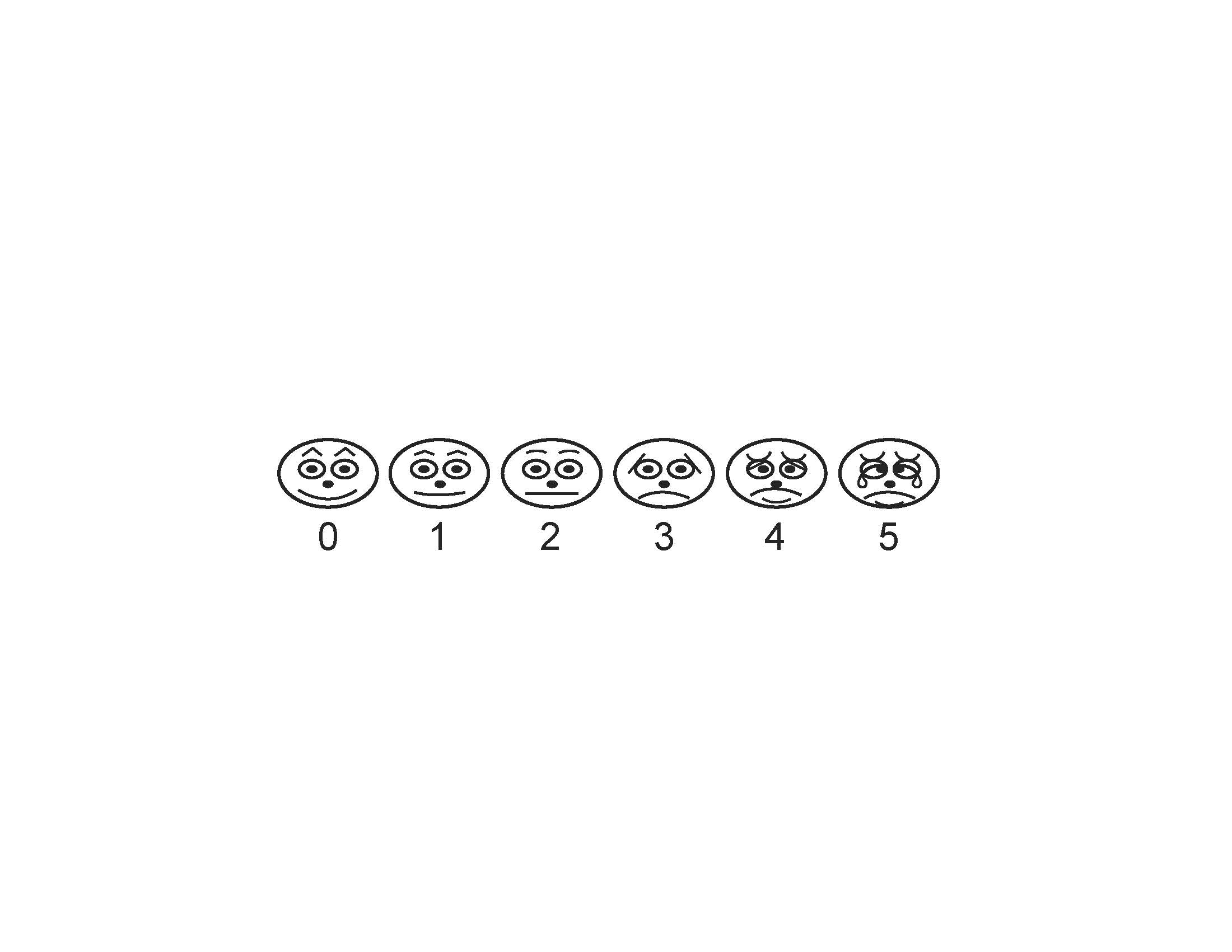


**Figure S4. ESR Levels**


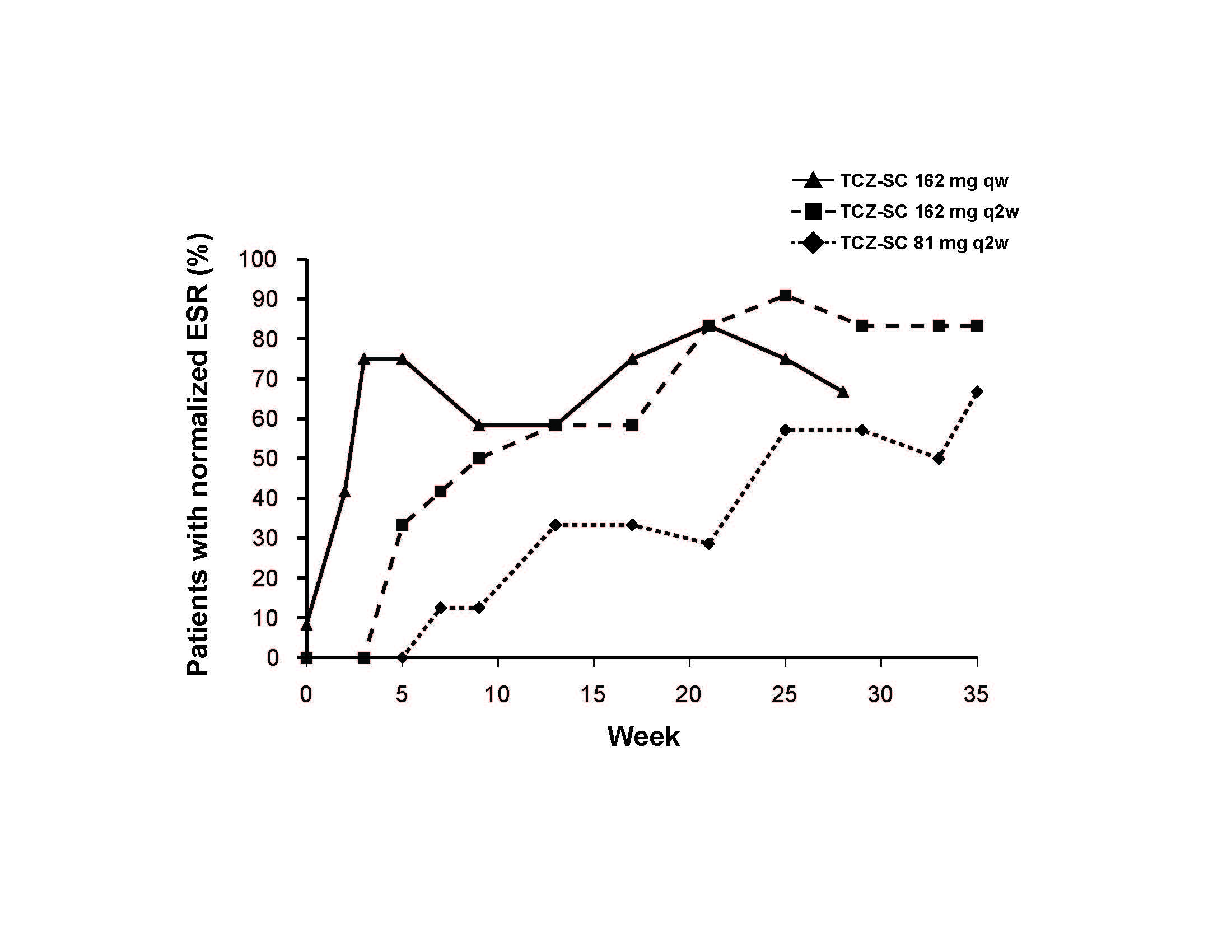


ESR, erythrocyte sedimentation rate.
